# Disseminated lymph node tuberculosis after splenectomy: an unusual case report in an adolescent

**DOI:** 10.1186/s12879-021-06859-1

**Published:** 2021-11-24

**Authors:** Moxin Cheng, Yu Chen

**Affiliations:** Department of Pediatric Tuberculosis, Shenyang Tenth People’s Hospital, Shenyang Chest Hospital, Liaoning 110044 Shenyang, China

**Keywords:** Adolescent, Splenectomy, Disseminated tuberculous lymphadenitis

## Abstract

**Background:**

Splenectomized patients are at an increased risk for overwhelming post-splenectomy infections typically with encapsulated bacteria. The clinical association between splenectomy and lymph-node tuberculosis is unclear.

**Case presentation:**

We describe a rare case of disseminated tuberculous lymphadenitis in an 18-year-old woman with history of splenectomy because of hereditary sherocytosis. She was admitted with enlargement of bilateral-cervical and left-axillary lymph nodes and fever. A diagnosis of probable tuberculosis was made based on the findings of fine-needle aspiration. Histology showed granulomas and extensive caseous necrosis, with the site of puncture located at an enlarged lymph node on the right side. The diagnosis was confirmed via nucleic-acid amplification tests following excisional biopsy of the left axillary lymph node. Disseminated tuberculous lymphadenitis was localized in the bilateral neck, right lung hilum, left sub-axillary region, and mediastinum, as detected from contrast-enhanced computed tomography of the neck.

**Conclusions:**

*Mycobacterium tuberculosis* infection should be considered in children and adolescents with extensive enlargement of lymph nodes after splenectomy.

## Background

Splenectomy is a possible treatment for splenic rupture, hypersplenism, “wandering spleen,” hereditary spherocytosis (HS), autoimmune hemolytic anemia, immune thrombocytopenia, lymphoma, and other hematological diseases in children [1]. HS is a rare genetic disease affecting erythrocyte membranes that leads to production of characteristically spherical erythrocytes (“spherocytes”). The estimated prevalence of HS worldwide is 0.05% (~1 in 2000 individuals). If the degree of anemia is extremely high, surgical management of HS *via* splenectomy is the only feasible intervention [2]. However, post-splenectomy infection is a considerable challenge, and encompasses a wide spectrum of infectious processes. Post-splenectomy sepsis caused by pneumococcal, *Haemophilus influenzae*, or *Neisseria meningitidis* infections has been described extensively [3]. Lai et al. [4] investigated the relationship between splenectomy and tuberculosis. They found that splenectomized patients had a 1.9-fold increased risk of pulmonary tuberculosis, with a 4.8-fold increased risk for those who underwent splenectomy but had comorbidities such as chronic obstructive pulmonary disease. Tuberculous meningitis caused by dissemination of *Mycobacterium tuberculosis* after splenectomy in adults has been reported [5]. However, studies on disseminated lymphatic tuberculosis after splenectomy in children and adolescents have not been documented.

Tuberculous lymphadenitis is the most common extrapulmonary manifestation of tuberculosis [6]. Disseminated tuberculous lymphadenitis due to *M. tuberculosis* infection can be serious, and highlights the importance of a timely diagnosis and efficacious therapeutic strategies.

With the permission of the ethics review board of Shenyang Tenth People’s Hospital (Shenyang, China), we describe a rare case of an 18-year-old patient presenting with disseminated lymph-node tuberculosis after splenectomy.

### Case presentation

This case report is based on an 18-year-old Chinese woman diagnosed with HS 2 years previously who had a paternal history of HS. Signs and symptoms were splenomegaly, severe anemia, jaundice, reticulocytosis, fatigue, and abdominal discomfort. Total splenectomy (TS) was undertaken through a laparotomy incision. Before elective splenectomy, an antibiotic (latamoxef 250 mg, i.v., b.i.d., 1 week) was used prophylactically to reduce the risk of potentially fatal overwhelming post-splenectomy sepsis (OPSS) from encapsulated bacteria. After TS, the patient was in good health. No evidence of infection with pneumococci, *H. influenzae*, *N. meningitides*, or other bacteria was found. The patient was not screened for *M. tub*e*rculosis* infection until the present time. The patient had received the bacillus Calmette–Guérin vaccine as a child in China, and did not have a history of contact with a person with active tuberculosis.

Initially, the patient experienced fever, fatigue, and weight loss for 5 months before presentation, with subsequent development of enlargement of cervical lymph nodes for 1 week. Color ultrasound images showed enlargement of multiple lymph nodes (1.51 cm × 0.52 cm in zone II of the neck on the right side; 2.75 cm × 1.66 cm in zone IV of the neck on the right side; 1.92 cm × 0.71 cm in zone II of the neck on the left side; 1.11 cm × 1.00 cm in the right supraclavicular fossa; 1.00 cm × 0.66 cm in the left supraclavicular fossa). The patient was screened for suspected hematological diseases (e.g., malignant lymphoma) through puncture biopsy of lymph nodes by the Hematology Department of Shengjing Hospital of China Medical University (a general hospital in Shenyang). In addition, the histological features of the enlarged right cervical lymph node revealed granulomatous lesions with necrosis. Further examination of the lungs using computed tomography (CT) disclosed that the right hilar lymph node was enlarged and adjacent bronchi were compressed and narrowed, along with enlarged lymph nodes in the mediastinum and left axillary region. These findings pointed towards a diagnosis of disseminated tuberculous lymphadenitis.

In January 2021, the patient was admitted to the Department of Pediatric Tuberculosis within Shenyang Chest Hospital in Shenyang. Upon admission, the patient presented with fever, fatigue, respiratory rate of 19 breaths/min, and heart rate of 90 beats/min. Oxygen saturation in room air was normal. Blood pressure was 105/76 mmHg. Enlarged lymph nodes were found in the bilateral cervical regions and left axilla during physical examination. Lymph nodes in the right cervical region and left axilla were fluctuant, whereas those in the left cervical region were hard. All detected lymph nodes were solitary. Significant abnormal findings were not observed in other examinations.

Contrast-enhanced CT of the neck revealed a round, soft-tissue shadow (25.1 mm × 14.2 mm) in the right supraclavicular area with a CT value of 46 HU. Contrast-enhanced imaging revealed annular enhancement with a higher CT value of 70 HU. The right internal jugular vein was compressed and flat.

**Fig. 1:**
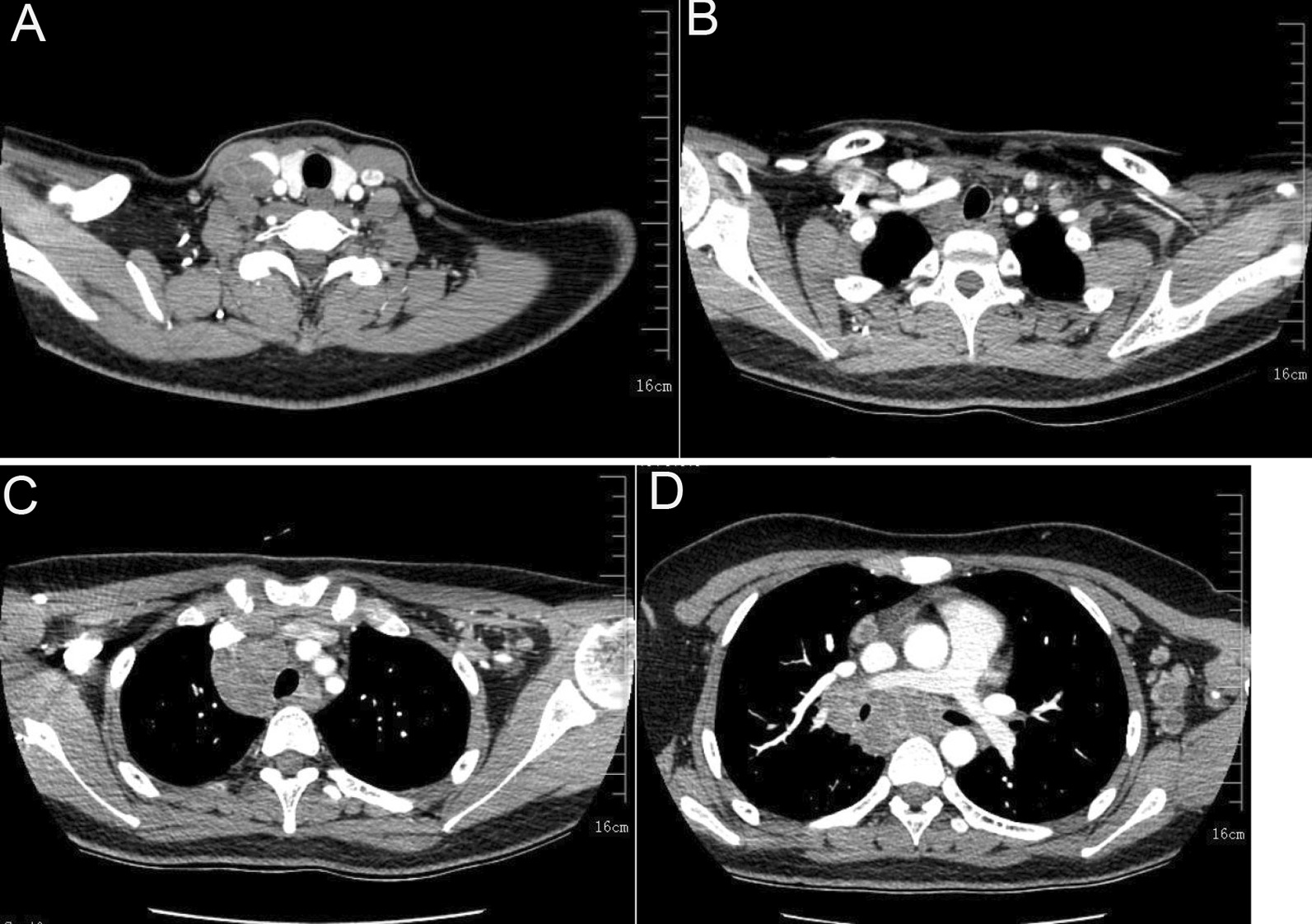
Contrast-enhanced CT of the neck. **A** Liquefaction in the adhesion and fusion regions of cervical lymph nodes. **B** Right supraclavicular lymph nodes with necrosis in their center. **C** Necrosis in the adhesion and fusion regions of anterior mediastinal lymph nodes. **D** Uneven density in the adhesion and fusion region of posterior mediastinal lymph nodes

Multiple enlarged lymph nodes (the largest sized 27.1 mm × 23.3 mm) were observed in the mediastinal space, thoracic entrance, and left axilla. Inhomogeneous annular enhancement was observed with a CT value of 36 HU. A lymph node of size 28.2 mm × 26.5 mm was detected in the right hilum with a CT value of 40 HU. Contrast-enhanced CT revealed inhomogeneous annular enhancement with a higher CT value of 65 HU (Fig. 1A–D). Color ultrasound images showed multiple enlarged lymph nodes in the bilateral neck, left axilla, and bilateral groin. The number, distribution, diameter, and manifestation of enlarged lymph nodes are presented in Table 1.

**Table 1:** Number, distribution, diameter, and CT findings of enlarged lymph nodes in different regions

Region	Number (count)	Size of lymph node (mm), short-axis diameter × long-axis diameter	CT value (plain/contrast-enhanced)	CT findings
Right cervical region	4–5	24.1 × 12.3	71/89	Liquefaction in the adhesion and fusion regions of cervical lymph nodes
Right supraclavicular region	1	25.1 × 14.2	46/70	Necrosis in the center of the lymph node
Anterior mediastinum	>10	40.1 × 36.6	40/86	Necrosis in the adhesion and fusion regions of lymph nodes
Posterior mediastinum	>10	37.2 × 47.3	48/95	Inhomogeneous density in the adhesion and fusion regions of lymph nodes
Left axilla	5–6	27.1 × 23.3	36/70	Necrosis in the adhesion and fusion regions of lymph nodes

The X.DOT-TB (TB Healthcare, Foshan, China) tuberculosis test result was positive (Table 2). The Xpert MTB/RIF test (Cepheid, Sunnyvale, CA, USA) result of the sputum specimen was negative. An induced sputum acid-fast bacillus smear was examined thrice, and was negative on each occasion. MGIT 960 culture of the sputum specimen (Becton Dickinson, Franklin Lakes, NJ, USA) was negative, along with the remainder of an extensive workup of infection detection. Subsequently, the patient was started on a four-drug regimen incorporating rifampin, isoniazid, pyrazinamide, and ethambutol for treatment of clinically diagnosed active tuberculous lymphadenitis. Results of laboratory examinations upon hospital admission are shown in Table 3.

**Table 2:** Result of X.DOT-TB

Negative control	ESAT6-CFP10	Positive control	Diagnosis
0	124	354	Positive

Positive responses were defined as ≥11 spots for the X.DOT-TB kit

**Table 3:** Summary of the results of laboratory findings

Blood tests(unit)	Result	Reference value
WBC (10^9^/L)	9.41	3.50–9.50
NEU%	62.3	40–75
HGB (G/L)	126	115–150
Plt (10^9^/L)	616	350
C reactive protein (mg/L)	17.8	0–4
PCT (ng/mL)	0.064	0.02–0.05
AST (U/L)	21	13–35
ALT (U/L)	20	7–40
Hiv TP	Negative	Negative
ESR (mm/L)	75	0-20
TSH (uIU/mL)	3.310	0.27–4.20
FT4 (pmol/L)	12.5	12–22
FT3 (pmol/L)	4.87	3.10–6.80
The total T-cell level (cells/µL)	2033	742–2750
The total T-cell proportion (%)	63%	50–84
The level of T-helper cells (cells/µL)	1140	500–1500
T-helper cells proportion (%)	35	27–51
The CD4:CD8 ratio	0.98	2.57
The number of B cells (cells/µL)	875	80–616
B cells proportion (%)	127	5–22

**Fig. 2:**
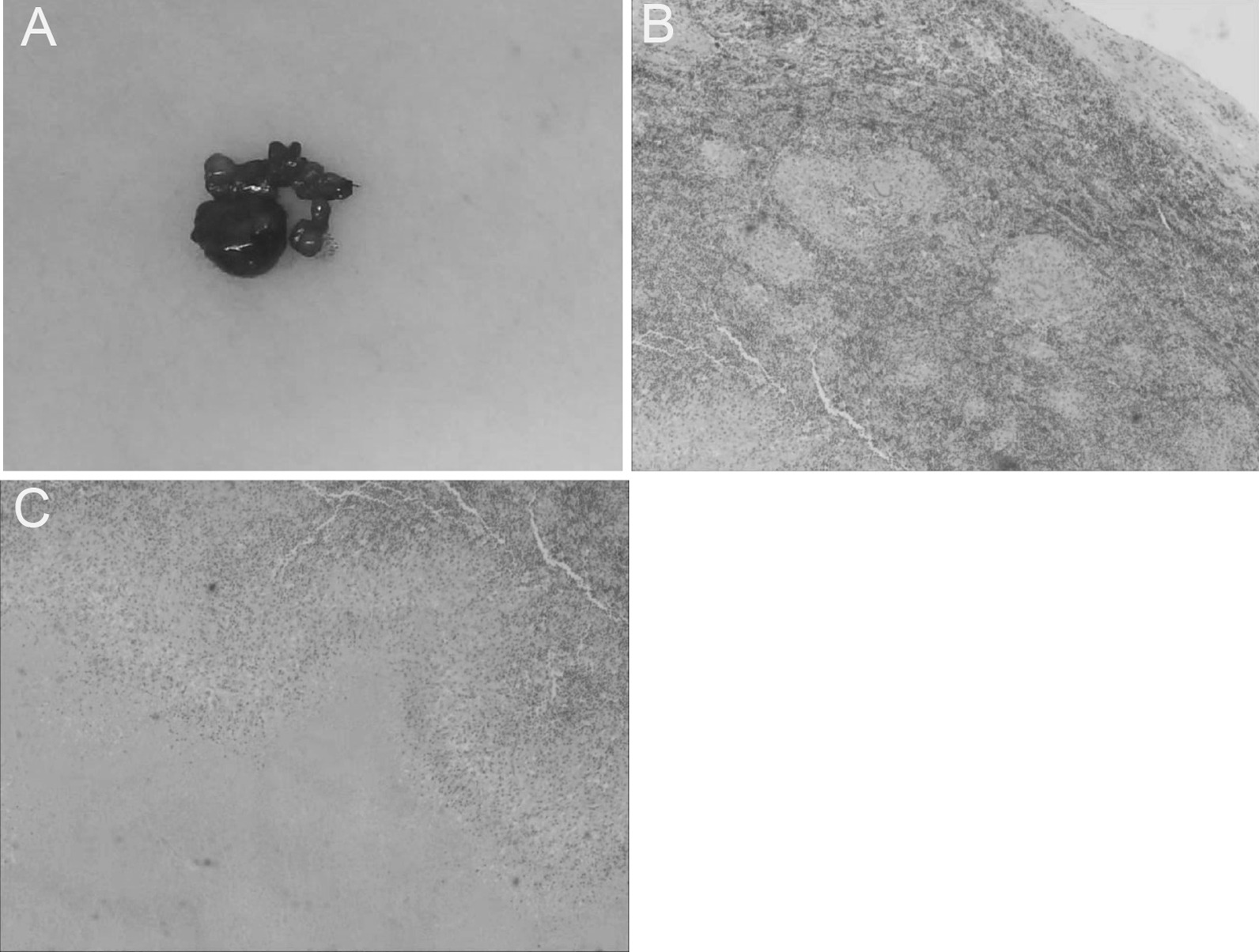
Histopathology image of the resected left axillary lymph node. **A** Multiple pieces of adipoid tissue in which nodules can be visualized, with the largest being approximately 3.0 cm × 2.0 cm × 1.7 cm and the smallest being ~1.3 cm in diameter. The capsule is relatively complete and caseous necrosis is evident. **B**,**C** Some tissues in lymph nodes were destroyed and replaced with pink-stained focal nonstructural necrosis surrounded by epithelioid and multinucleated giant cells

During the first 2 weeks of tuberculosis treatment, the patient complained of persistent pain in the left axillary lymph node. An enlarged lymph node was detected, and the left axillary lymph node was surgically excised (Fig. 2A). Lymphoid tissue was of dimension 3.0 cm × 2.0 cm × 1.7 cm. Postoperative pathology revealed granulomatous inflammation and caseous necrosis surrounded by epithelial-like and multinucleated giant cells (Fig. 2B, C). Results from the Xpert MTB/RIF assay of lymphoid-tissue specimens were positive. Lymphoid tissue was sensitive to rifampicin, which further confirmed the diagnosis of extensively disseminated *M. tuberculosis* into the lymph nodes. After receiving standard treatment for tuberculosis for 2 months, her clinical symptoms improved, and fever, fatigue, and emaciation disappeared. Enlarged lymph nodes in the neck, axilla, and groin were reduced. The patient continues to receive tuberculosis treatment. At this time, her clinical condition is stable.

## Discussion and conclusion

HS is a genetic disorder runs in the family that patients usually presenting with spherocyte and hematolysis because of mutations to cytoskeletal proteins that results in weakened cell membrane integrity [7]. Children with a family history of genetic disorders are more likely to develop HS. Symptoms, such as splenomegaly, severe anemia, and jaundice are caused by hemolysis of immature reticulocytes undergoing conditioning in the spleen, and severe anemia always calls for surgical removal of the spleen. According to family genetic history, anemia, jaundice, splenomegaly, and other signs in our patient, combined with a positive test for vascular fragility, hereditary spherocytosis was diagnosed. The patient underwent TS due to progressive anemia and severe splenomegaly. The spleen has immunological functions and protects the body against pathogen infections. Thus, TS is associated with increased long-term susceptibility to potentially fatal OPSS from encapsulated bacteria, such as pneumococci, *N. meningitides*, *H. influenzae*, and staphylococci. It has been reported that 3.2% of TS patients develop pathogen infection, and the overall mortality is 1.4%. The mortality prevalence associated with OPSS within 48 h after onset is 50–70% [8, 9]. Overall, 20% of cases of fatal sepsis occur within the first 6 months and 60% cases are recorded within 2 years after splenectomy [1]. The risk of OPSS from these organisms may be reduced with the use of prophylactic vaccinations and antibiotics before elective splenectomy, Dahyot-Fizelier et al. proposed that pneumococcal, *H. influenzae* type-b and meningococcal vaccination should be administered 2–6 weeks before scheduled splenectomy, and 2 weeks after urgent surgery [10]. Our patient was administered a broad-spectrum antibiotic (latamoxef) for 1 week following surgery, and pathogenic bacterial infections were not detected over the subsequent 2 years. However, lymphatic dissemination of *M. tuberculosis* after TS is commonly overlooked by clinicians, with only a few cases documented in the literature [4, 11]. This is the first reported case of disseminated lymphatic tuberculosis in an adolescent after splenectomy.

The spleen is a major lymphoid organ in the body responsible for generating antibodies as well as removing bacteria and aged, antibody-coated and damaged blood cells. The spleen contains many immune-competent cells: macrophages, T- and B-lymphocytes, natural killer cells, and dendritic cells. Furthermore, the spleen produces opsonins, complements, and endogenous cytotoxic factors. The spleen was proved to exert extensive usefulness in phagocytic activities as well as humoral immune responses, both of which would be largely repressed after a splenectomy [12]. Although the immune response against tuberculosis is dependent mostly on cell-mediated immunity, mounting evidence have suggested that B cell might have shoulder a critical role in anti-M. tuberculosis immunity. It might execute direct defend against M. tuberculosis as a frontline effector or redirect the effects of T cell-memory response as an intermediate. In a similar vein, other subsets of innate immune cells with prior ambiguous capacity to mount secondary “memory-like” responses are also potential arsenal in curbing M. tuberculosis proliferation and disseminating [13]. Similarly, different subpopulations of innate immune cells that possess a potential capacity to mount secondary “memory-like” responses are equally capable of limiting the growth of *M. tuberculosis* [14, 15]. As we reckon, splenectomy might also cause immune function impairment and distort our system to a state susceptible for tuberculosis development. The effect of TS on immune function has been investigated extensively. Borgers and colleagues collected peripheral blood before and after splenectomy to carry out single-cell cytometry based on time-of-flight mass spectrometry [16]. They showed that splenectomy induced significant long-term changes in the populations and functions of circulating immune cells. Patients infected with the human immunodeficiency virus (HIV), adult and child contacts of people with pulmonary tuberculosis, children younger than 5 years of age and severely malnourished children, as well as patients undergoing treatment with anti-tumor necrosis factor, receiving dialysis, preparing for organ or hematologic transplantation or with silicosis, are considered at-risk populations for progression from latent tuberculosis infection to active disease. The World Health Organization recommends screening for latent tuberculosis infection and early intervention in such populations, especially in countries with a high burden of tuberculosis. However, splenectomy has not been validated as a high-risk factor for tuberculosis susceptibility.

We focused on this potential link in clinical practice over a period of 2 years following surgery in our patient [1]. Following TS, the patient did not undergo screening for *M. tuberculosis* infection until the appearance of clinical signs (e.g., fever and enlargement of lymph nodes). Contrast-enhanced CT and color Doppler ultrasound images revealed enlargement of bilateral-cervical, left-axillary, and bilateral-groin lymph nodes. The X.DOT-TB tuberculosis test result was positive. Xpert MTB/RIF testing of pathologic tissue from the left axillary lymph node confirmed the diagnosis of disseminated tuberculosis. In this case, immunosuppression after TS promoted the occurrence and continuous progression of extensively disseminated *M. tuberculosis* to lymph nodes. Unfortunately, our patient did not undergo screening for *M. tuberculosis* infection or receive preventive treatment appropriate for a high-risk group. In view of these findings, children and adolescents subjected to splenectomy should be considered a population of significant risk for *M. tuberculosis* infection and disease progression, and require active treatment responses.

Tuberculous lymphadenitis was recognized as an exclusive pathological lesion caused by bacteria of the *M. tuberculosis* complex. In most series, tuberculous lymphadenitis has been shown to be more common among women than men. Although previously considered to be a disease in children, the peak age range documented recently has been 30–40 years. Several risk factors are related to the development of active tuberculous lymphadenitis. HIV infection is the biggest risk factor, with malnutrition, alcoholism, diabetes mellitus, and smoking identified as other important risk factors [17]. Other reported risk factors are hematologic malignancies, silicosis, gastrectomy, and treatment with corticosteroids or immunosuppressive medications [18]. Our findings support the inclusion of splenectomy as an potential risk factor for tuberculous lymphadenitis.

*M. tuberculosis* infection leads to the formation, expansion, and destruction of granulomas in lymph nodes, which can replace normal structures that have important functions. A granuloma is a tiny cluster of white blood cells and other tissue that can be found in parts of the body in some people. A granuloma plays a key part in organizing interactions between immune cells. This action leads to an effective response that inhibits and kills bacilli, contains the infection, prevents spread, and localizes the inflammatory response and tissue damage [19]. Owing to the influence of splenectomy on cellular immune function, a granuloma is not limited to lymph nodes. *M. tuberculosis* can use a granuloma as an expanding medium to spread among cells, which leads to transmission to lymph nodes in other parts of the body. The epidemiologic characteristics of tuberculous lymphadenitis differ from those of pulmonary tuberculosis, exhibit variable clinical manifestations, and no adequate gold-standard test. so the diagnosis can be a considerable challenge. Recently developed ELISpot are sensitive, specific, and rapid immunodiagnostic tests for TB infection. They detect interferon-γ (IFN-γ) produced by lymphocytes in response to Mycobacterium tuberculosis (MTB)-specific antigens, early secretory antigenic target 6 (ESAT-6), and culture filtrate protein-10 (CFP-10). Immunoassays capable of detecting the host’s immune response specific to TB causative agent Mycobacterium Tuberculosis (M.TB) has become an alternative diagnostic aid for tuberculous lymphadenitis. A definitive diagnosis of tuberculous lymphadenitis can be made *via* culture or detection using polymerase chain reactions of *M. tuberculosis* in affected lymph nodes, thereby facilitating differentiation from other mycobacteria that may cause lymphadenitis. Histologic features, such as non-specific lymphoid infiltrates, non-caseating granulomas, or the presence of Langerhans giant cells in areas of extensive caseous necrosis, support the diagnosis of probable tuberculosis in acid-fast bacillus-negative, culture-negative cases. Excisional biopsy is the most invasive approach for the diagnosis, but has the highest sensitivity. Lau et al. [20] also confirmed the high sensitivity in that they found a nearly 4-time higher positive rate when comparing the culture results between excisional biopsy and fine-needle aspiration. Importantly for clinicians, the anti-TB treatment could be deflated since the response to medication might be obtuse and patients might present recurrent or new lymph nodes enlargement during and after previously efficacious treatment. During treatment for tuberculosis, our patient complained of persistent pain in the left enlarged axillary lymph node and subsequently underwent left axillary lymphadenectomy. Postoperative pathology revealed the typical changes of tuberculous granulomas. The diagnosis of disseminated tuberculosis to the lymph nodes was confirmed *via* Xpert MTB/RIF testing of lymphoid tissue. Excisional biopsy could deliver an instant and comforting symptomatic responses, and is chosen as an priori treatment especially for those complicated with multiple nodes [21, 22]. Our patient clearly benefited from excisional biopsy.

## Conclusions

*M. tuberculosis* infection after splenectomy should be considered in children and adolescents with enlarged lymph nodes. Children who have undergone splenectomy should be considered a high-risk population and prioritized for screening of *M. tuberculosis* infection. While the diagnosis of tuberculous lymphadenitis is challenging, the treatment might be even more complex since many cases developed recurrent lymph nodes swelling even during or after previously efficacious treatment. Excisional biopsy and PCR-based detection of *M. tuberc*ulosis should be undertaken early to confirm the diagnosis of tuberculous lymphadenitis.

## Data Availability

All data and material collected during this study are available from the corresponding author upon reasonable request.

## References

[1] Weledji EP (2014). Benefits and risks of splenectomy. Int J Surg.

[2] Guizzetti L (2016). Total versus partial splenectomy in pediatric hereditary spherocytosis: A systematic review and meta-analysis. Pediatr Blood Cancer.

[3] Ellison EC, Fabri PJ (1983). Complications of splenectomy, etiology, prevention, and management. Surg Clin North Am.

[4] Lai SW, Wang IK, Lin CL, Chen HJ, Liao KF (2014). Splenectomy correlates with increased risk of pulmonary tuberculosis: a case-control study in Taiwan. Clin Microbiol Infect.

[5] Sheng CF, Liu BY, Zhang HM, Zheng X (2015). Overwhelming postsplenectomy infection. Genet Mol Res.

[6] Cataño JC, Robledo J. Tuberculous lymphadenitis and parotitis. Microbiol Spectr. 2016;4. doi:10.1128/microbiolspec.TNMI7-0008-2016. 10.1128/microbiolspec.TNMI7-0008-2016 28084205

[7] Bolton-Maggs PH, Langer JC, Iolascon A, Tittensor P, King MJ (2012). General Haematology Task Force of the British Committee for Standards in Haematology. Guidelines for the diagnosis and management of hereditary spherocytosis: 2011 update. Br J Haematol.

[8] Bisharat N, Omari H, Lavi I, Raz R (2001). Risk of infection and death among post- splenectomy patients. J Infect.

[9] Okabayashi T, Hanazaki K (2011). Overwhelming postsplenectomy infection syndrome in adults—a clinically preventable disease. Br J Haematol.

[10] Dahyot-Fizelier C, Debaene B, Mimoz O (2013). Gestion du risque infectieux chez le splénectomisé [Management of infection risk in asplenic patients]. Ann Fr Anesth Reanim.

[11] Niblock AL (2011). Recurrent neck abscesses due to cervical tuberculous lymphadenopathy in an elderly woman post-splenectomy: a case report. J Med Case Rep.

[12] Altamura M, Caradonna L, Amati L, Pellegrino NM, Urgesi G, Miniello S (2001). Splenectomy and sepsis: the role of the spleen in the immune-mediated bacterial clearance. Immunopharmacol Immunotoxicol.

[13] Rao M, Valentini D, Poiret T, Dodoo E, Parida S, Zumla A, Brighenti S, Maeurer M (2015). B in TB: B cells as mediators of clinically relevant immune responses in tuberculosis. Clin Infect Dis.

[14] Venkatasubramanian S, Cheekatla S, Paidipally P, Tripathi D, Welch E, Tvinnereim AR (2017). IL-21-dependent expansion of memory-like NK cells enhances protective immune responses against Mycobacterium tuberculosis. Mucosal Immunol.

[15] Kaufmann E, Sanz J, Dunn JL, Khan N, Mendonca LE, Pacis A (2018). BCG educates hematopoietic stem cells to generate protective innate immunity against tuberculosis. Cell.

[16] Borgers JSW, Tobin RP, Vorwald VM, Smith JM, Davis DM (2020). High-Dimensional Analysis of Postsplenectomy Peripheral Immune Cell Changes. lmmunoHorizons.

[17] Morán-Mendoza O, Marion SA, Elwood K, Patrick D, FitzGerald JM (2010). Risk factors for developing tuberculosis: a 12-year follow-up of contacts of tuberculosis cases. Int J Tuberc Lung Dis.

[18] Lönnroth K, Castro KG, Chakaya JM, Chauhan LS, Floyd K, Glaziou P, Raviglione MC (2010). Tuberculosis control and elimination 2010–50: cure, care, and social development. Lancet.

[19] Dye C, Williams BG (2010). The population dynamics and control of tuberculosis. Science.

[20] Lau SK, Wei WI, Hsu C, Engzell UC (1990). Efficacy of fine needle aspiration cytology in the diagnosis of tuberculous cervical lymphadenopathy. J Laryngol Otol.

[21] Artenstein AW, Kim JH, Williams WJ, Chung RC (1995). Isolated peripheral tuberculous lymphadenitis in adults: current clinical and diagnostic issues. Clin Infect Dis.

[22] Blaikley JF, Khalid S, Ormerod LP (2011). Management of peripheral lymph node tuberculosis in routine practice: an unselected 10-year cohort. Int J Tuberc Lung Dis.

